# Development and Validation of a Website to Guide Decision-Making for Disorders of Consciousness

**DOI:** 10.3389/fnagi.2022.934283

**Published:** 2022-07-07

**Authors:** Junwei Kang, Yuan Zhong, Gengfa Chen, Lianghua Huang, Yunliang Tang, Wen Ye, Zhen Feng

**Affiliations:** Department of Rehabilitation Medicine, First Affiliated Hospital of Nanchang University, Nanchang, China

**Keywords:** clinical prediction, nomogram, Glasgow coma scale score, disorders of consciousness (DOC), website

## Abstract

**Background:**

This study aimed to develop and validate a nomogram and present it on a website to be used to predict the overall survival at 16, 32, and 48 months in patients with prolonged disorder of consciousness (pDOC).

**Methods:**

We retrospectively analyzed the data of 381 patients with pDOC at two centers. The data were randomly divided into training and validation sets using a ratio of 6:4. On the training set, Cox proportional hazard analyses were used to identify the predictive variables. In the training set, two models were screened by COX regression analysis, and based on clinical evidence, model 2 was eventually selected in the nomogram after comparing the receiver operating characteristic (ROC) of the two models. In the training and validation sets, ROC curves, calibration curves, and decision curve analysis (DCA) curves were utilized to measure discrimination, calibration, and clinical efficacy, respectively.

**Results:**

The final model included age, Glasgow coma scale (GCS) score, serum albumin level, and computed tomography (CT) midline shift, all of which had a significant effect on survival after DOCs. For the 16-, 32-, and 48-month survival on the training set, the model had good discriminative power, with areas under the curve (AUCs) of 0.791, 0.760, and 0.886, respectively. For the validation set, the AUCs for the 16-, 32-, and 48-month survival predictions were 0.806, 0.789, and 0.867, respectively. Model performance was good for both the training and validation sets according to calibration plots and DCA.

**Conclusion:**

We developed an accurate, efficient nomogram, and a corresponding website based on four correlated factors to help clinicians improve their assessment of patient outcomes and help personalize the treatment process and clinical decisions.

## Introduction

The treatment of disorders of consciousness (DOCs) is a global challenge. Prolonged DOC (pDOC) refers to the loss of consciousness for more than 28 days, which usually occurs after traumatic brain injury, especially when severe (Kondziella et al., 2020). There is currently no effective treatment for DOCs, and most patients are bedridden and require long-term and arduous care. The cost of treatment and care from injury to death may be as high as US$1 million (Ragnarsson, 2002). Globally, the number of patients with pDOC is staggering. Reportedly, there are ~100,000–300,000 patients with pDOC in the United States (Giacino et al., 2018), whereas in Europe, the proportion is about 0.2–6.1 cases for every 100,000 people (Erp et al., 2015). No accurate reports exist on the matter in China, but it is commonly thought that the incidence and prevalence of pDOC are increasing. This poses a challenge for clinical medicine and places a large burden on the local economy. The prognosis of DOC is directly related to the decisions made by the patients' family members and clinicians, which form the basis for further neurological rehabilitation. Therefore, objective and sound prognostication is important and urgently needed. However, accurately predicting the outcomes of patients with DOCs remains a challenge.

At present, evaluation of the clinical prognosis of patients with DOC mainly relies on scales and clinical manifestations, but this has disadvantages, such as high subjectivity and poor accuracy. Song et al. (2018) studied the relationship between resting-state cerebral functional MRI (fMRI) and the 1-year outcome of patients with pDOC. Certain patterns found in the sleep electroencephalogram (EEG) can be used to predict the short-term outcome of pDOC patients (Yang et al., 2020). Anderson et al. (2020) found that three biomarkers (GFAP, UCH-L1, and MAP-2) were related to the recovery of patients with pDOC within 6 months. Unfortunately, today no universally applicable scoring system for predicting mortality in patients with DOCs is available. Therefore, the goal of our study was to develop and validate a system for scoring the risk of death in patients with pDOC. Such a system can help to identify patients at high risk of death, which, in turn, will contribute to their clinical management.

The characteristic of a nomogram is that it contains many variables and can predict the probability of an event in a single patient (Kattan, 2002). In this study, we developed a website to predict the survival rate of patients with pDOC based on a nomogram. In addition, two data sets were used to evaluate the performance, discrimination, and calibration of the model. The model is simple and practical, and can be used as an early warning and prediction system for patients with DOC.

## Methods

### Study Participants

This study involved participants from two research centers, the First Affiliated Hospital of Nanchang University and Shangrao Hospital of Traditional Chinese Medicine. The keywords, “coma” and “disorder of consciousness,” were entered into the medical record information system, and all the patients admitted from January 1, 2016, to January 1, 2021, who met these conditions were considered. Patients with pDOC caused by anoxic, traumatic, or vascular events, and with a course of at least 28 days (Giacino et al., 2018), were included in this study.

Patients with a history of craniocerebral injury or DOC not caused by craniocerebral injury, as well as those without follow-up data, information on cause of death, and incomplete medical records were excluded from this study. A total of 381 patients were finally included and randomized into training (60%) and validation (40%) cohorts.

### Data Collection

Seventeen potential predictors were collected in detail from the electronic medical records of the selected patients, including baseline demographic data (age, sex, and etiology such as trauma, stroke, or hypoxia), patient status at admission [state of consciousness such as vegetative state (VS) or minimally concious state (MCS), Glasgow coma scale (GCS) and Coma Recovery Scale-Revised (CRS-R) scores, and pupillary light reflex], laboratory test results (albumin and lateral shift of cerebral midline structures), complications (epilepsy, hydrocephalus, cobwebs, and submembranous hemorrhage), and medical history (hypertension, diabetes, smoking history, multiple trauma, and history of craniotomy). The duration of survival and survival status of each patient were also extracted. To reduce sampling bias, data were obtained with consultation, involving effective communication and cross-checking, of the medical staff.

### Statistical Analysis

In order to improve the accuracy of our statistical analysis, and to reduce the bias caused by missing data, this paper made multiple data imputations (Sterne et al., 2009). Patients were randomly divided into training and validation cohorts using a ratio of 6:4. The comparability of the two groups was assessed (Table 1). Continuous data conforming to the normal distribution were presented as mean ± standard deviation (m ± s), and independent samples *t*-tests were used to infer differences between the training and validation sets. For those with skewed distribution, the median (1st quartile, 3rd quartile) was used to describe them, and the Mann-Whitney U test was used to compare the two groups. Categorical variables were expressed as frequencies (proportions), and the chi-squared test or Fisher's exact test was used for their comparison.

**Table 1:** Comparison of baseline data between the training set and validation set.

**Features**	**Training set**	**Validation set**	***P*-value**
	**(*n* = 229)**	**(*n* = 152)**	
Age (years)	52.14 ± 14.75	53.52 ± 15.04	0.374
**Sex**			0.629
Male	162 (70.7%)	104 (68.4%)	
Female	67 (29.3%)	48 (31.6%)	
**Level of consciousness**			0.492
VS	152 (66.4%)	106 (69.6%)	
MCS	77 (33.6%)	46 (30.4%)	
**Etiology**			0.743
Trauma	115 (50.2%)	72 (47.4%)	
Stroke	92 (40.2%)	67 (44.1%)	
Anoxia	22 (9.6%)	13 (8.5%)	
GCS total score	9.00 (6.00, 9.00)	8.00 (6.00, 9.00)	0.618
CRS-R total score	5.00 (3.00, 8.00)	5.00 (4.00, 8.00)	0.754
**Serum albumin**			0.326
≥35 g/L	147 (64.2%)	90 (59.2%)	
<35 g/L	82 (35.8%)	62 (40.8%)	
**Epilepsy**			0.685
Presence	24 (10.5%)	14 (9.2%)	
Absence	205 (89.5%)	138 (90.8%)	
**Hydrocephalus**			0.077
Presence	33 (14.4%)	12 (7.9%)	
Absence	196 (85.6%)	140 (92.1%)	
**Multiple injuries**			0.398
Presence	88 (38.4%)	65 (42.8%)	
Absence	141 (61.6%)	87 (57.2%)	
**Midline shift**			0.275
Presence	52 (22.7%)	42 (27.6%)	
Absence	177 (77.3%)	110 (72.4%)	
**Hypertension**			0.752
Presence	85 (37.1%)	54 (35.5%)	
Absence	144 (62.9%)	98 (64.5%)	
**Diabetes**			0.404
Presence	63	36	
Absence	166	116	
**Smoking history**			0.119
Presence	33 (%)	13 (%)	
Absence	196 (%)	139 (%)	
**Subarachnoid hemorrhage**			0.471
Presence	85	62	
Absence	144	90	
**Craniotomy**			0.739
Presence	106	73	
Absence	123	79	
**Pupillary light reflex**			0.507
One or both absent	56	30	
Presence	173	122	
**State**			0.332
Death	61	33	
Survival	168	119	

To screen predictors, we performed Cox proportional hazards analyses in the training set. Variables with a *p*-value of < 0.05 in the multivariate Cox regression were used as independent predictors (Abougergi et al., 2018). However, it is important to consider both clinical and statistical significance when selecting covariates for inclusion. Therefore, we used the state of consciousness with a *p*-value of >0.05 in the Cox regression analysis in our model, based on sufficient clinical evidence (Giacino et al., 2018). We combined variables with significant differences in the Cox regression and variables based on clinical evidence to develop two predictive models. In order to select the optimal model, Delong's test was used to compare the area under the receiver operating characteristic (ROC) curve of model 1 and model 2, and finally incorporate the optimal model into the nomogram. For both the training and validation groups, the performance of the nomogram was evaluated with calibration plots and decision curve analysis (DCA). All analyses were performed using R software (version 3.6.3).

Based on the scores for each factor in the nomogram, an overall risk score was calculated for each patient in the entire cohort. Patients were divided into two groups according to their risk: low and high risks (a restricted cubic spline was used to model the non-linear relationship between the overall risk score and the survival of patients with DOC). The Kaplan-Meier and log-rank methods were used to compare the survival of the two groups.

## Results

### Baseline Patient Characteristics

A total of 381 patients from the two centers were included. The general data of the training and validation cohorts are shown in Table 1. During the follow-up period, 61 deaths were recorded for the training cohort, representing a mortality rate of 26.6%. For the validation cohort, 33 deaths were recorded, representing a mortality rate of 21.7%.

### Development of Nomogram for DOC

**Figure 1:**
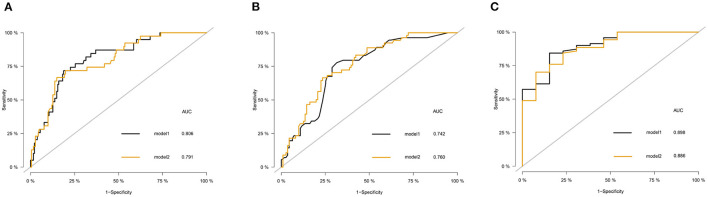
The receiver operating characteristic (ROC) curve of the model 1 and model 2 in the training set. **(A)** Comparison of ROC curves of model 1 and model 2 for predicting the survival of patients with disorder of consciousness (DOC) at 16 months. **(B)** Comparison of ROC curves of model 1 and model 2 for predicting the survival of patients with DOC at 32 months. **(C)** Comparison of ROC curves of model 1 and model 2 for predicting the survival of patients with DOC at 48 months.

**Figure 2:**
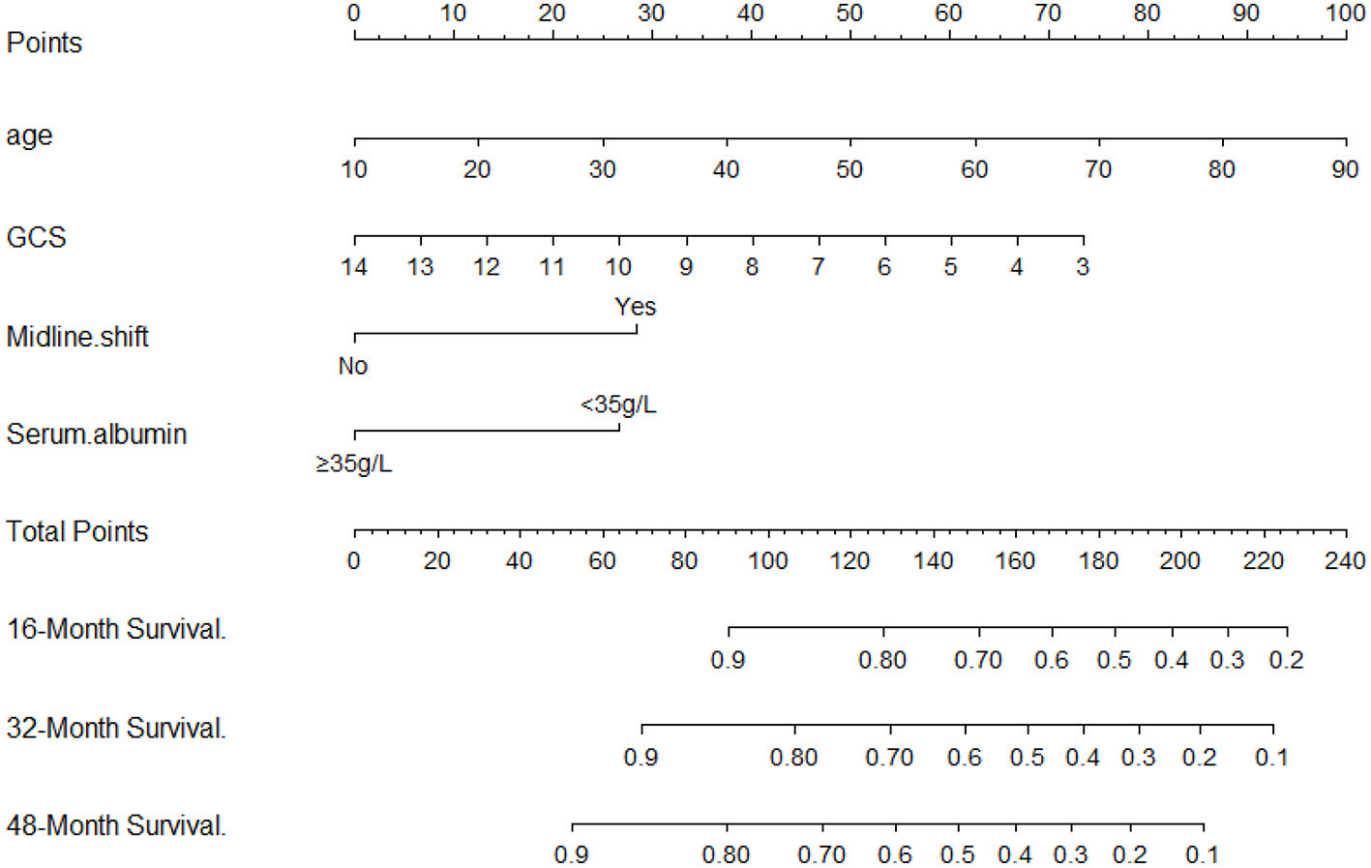
A clinical feature model was used to develop a nomogram.

Univariate analysis showed that age, GCS score, state of consciousness, diabetes, albumin, and computed tomography (CT) midline shift were associated with the risk of death from a DOC. Multivariate Cox regression analysis revealed that four of these variables (age, GCS score, CT midline shift, and albumin) were independent risk factors for DOC death (Table 2). The state of consciousness did not show a significant difference in the multivariate analysis. However, based on clinical evidence, we combined the variables with significant differences during Cox regression and variables based on clinical evidence to develop two predictive models—model 1: age + CT midline shift + albumin + GCS + state of consciousness; model 2: age + CT Midline shift + albumin + GCS. The individual performance and comprehensive performance of the two models were analyzed using ROC curves to determine which one was optimal. The ROC curves of models 1 and 2 at 16, 32, and 48 months were similar: 80.6 vs. 79.1, *p* = 0.075; 74.2 vs. 76, *p* = 0.162; and 89.8 vs. 88.6, *p* = 0.270, respectively (Figure 1). Since the best model should be the simplest and with fewer variables (Van, 2018), we selected the four variables of model 2 and incorporated them into the nomogram (Figure 2), which is an intuitive visual model. According to the nomogram, age had the greatest impact on DOC prognosis, followed by the GCS score, midline shift, and albumin. The total score was the sum of the individual scores of these four variables, and the specific probabilities of survival at 16, 32, and 48 months were finally derived based on the score.

**Table 2:** Cox regression analyses of prognostic factors in patients with prolonged disorders of consciousness in the training set.

**Variable**	**Univariate**		**Multivariate**	
	**HR (95%CI)**	***P*-value**	**HR (95%CI)**	***P*-value**
Age (years)	1.041 (1.022–1.061)	0.000	1.022 (1.002–1.044)	0.0305
**Sex**				
Female	Ref			
Male	0.787 (0.439–1.410)	0.421		
**Level of consciousness**				
VS	Ref		Ref	
MCS	0.386 (0.196–0.762)	0.006	0.525 (0.222–1.243)	0.143
**Etiology**				
Trauma	Ref			
Stroke	1.123 (0.657–1.918)	0.671		
Anoxia	1.509 (0.661–3.447)	0.328		
GCS total score	0.798 (0.717–0.889)	0.000	0.874 (0.783–0.976)	0.0162
CRS-R total score	0.819 (0.449–1.494)	0.514		
**Serum albumin(g/L)**				
<35	Ref		Ref	
≥35	0.434 (0.262–0.717)	0.001	0.580 (0.342–0.984)	0.0433
**Epilepsy**				
Presence	Ref			
Absence	1.363 (0.546–3.403)	0.507		
**Hydrocephalus**				
Presence	Ref			
Absence	0.554 (0.304–1.007)	0.053		
**Multiple injuries**				
Presence	Ref			
Absence	1.698 (0.970–2.974)	0.064		
**Midline shift**				
Presence	Ref		Ref	
Absence	0.417 (0.248–0.700)	0.001	0.557 (0.319–0.971)	0.039
**Hypertension**				
Presence	Ref			
Absence	0.622 (0.376–1.029)	0.065		
**Diabetes**				
Presence	Ref		Ref	
Absence	0.485 (0.290–0.813)	0.006	0.618 (0.366–1.045)	0.073
**Smoking history**				
Presence	Ref			
Absence	0.643 (0.342–1.209)	0.170		
**Pupillary light reflex**				
One or both absent	Ref			
Presence	0.644 (0.373–1.111)	0.114		
**Subarachnoid hemorrhage**				
Presence	Ref			
Absence	1.045 (0.619–1.763)	0.869		
**Craniotomy**				
Presence	Ref			
Absence	0.990 (0.599–1.637)	0.969		

### Establishment of a Website

**Figure 3:**
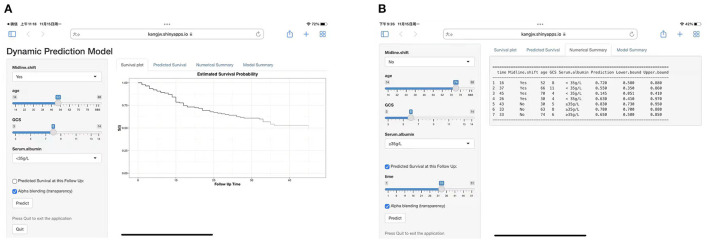
Construction of a web-based calculator for predicting DOC's disease survival based on the model (https://kangjw.shinyapps.io/P-Doc/). **(A)** Web survival rate calculator. **(B)** 95% confidence interval (CI) of the web survival rate calculator.

To facilitate the use of the nomogram in clinical settings, we built a website (https://kangjw.shinyapps.io/P-Doc/). For example: at 52 years of age, with midline shift, GCS score of 8, and albumin concentration of <35 g/l, the 16-month survival rate was ~72% [95% confidence interval (CI): 58–88.0%] (Figure 3).

### Model Performance on the Training Set

**Figure 4:**
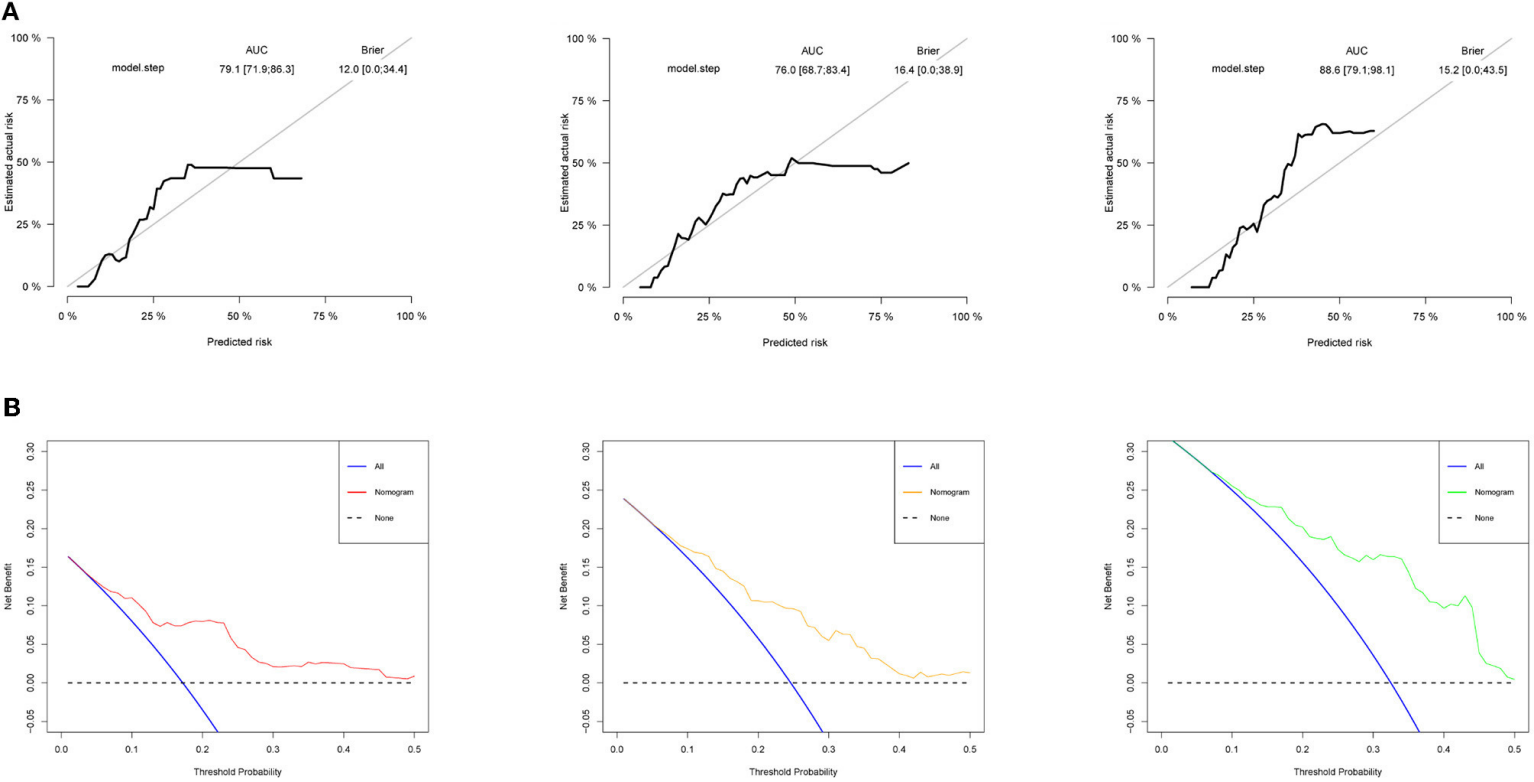
Model performance in the training set. **(A)** Calibration curves of 16-, 32-, and 48-month specific survival. **(B)** Decision curve analyses (DCA) for predicting the survival of patients with DOC at 16, 32, and 48 months by nomogram.

The ROC curve was used to evaluate the detection performance of the model in the training set. The area under the curve (AUC) values for predicting the 16-, 32-, and 48-month survival rates were 0.791, 0.760, and 0.886, respectively (Figure 1), indicating that the nomogram was effective at predicting prognosis. The calibration curve based on the training set showed that the nomogram had good calibration capabilities (Figure 4A). In addition, the DCA curves showed that the nomogram could be used for valuable judgments (Figure 4B). The management of patients with DOCs will benefit from this predictive model.

### Model Performance on the Validation Set

**Figure 5:**
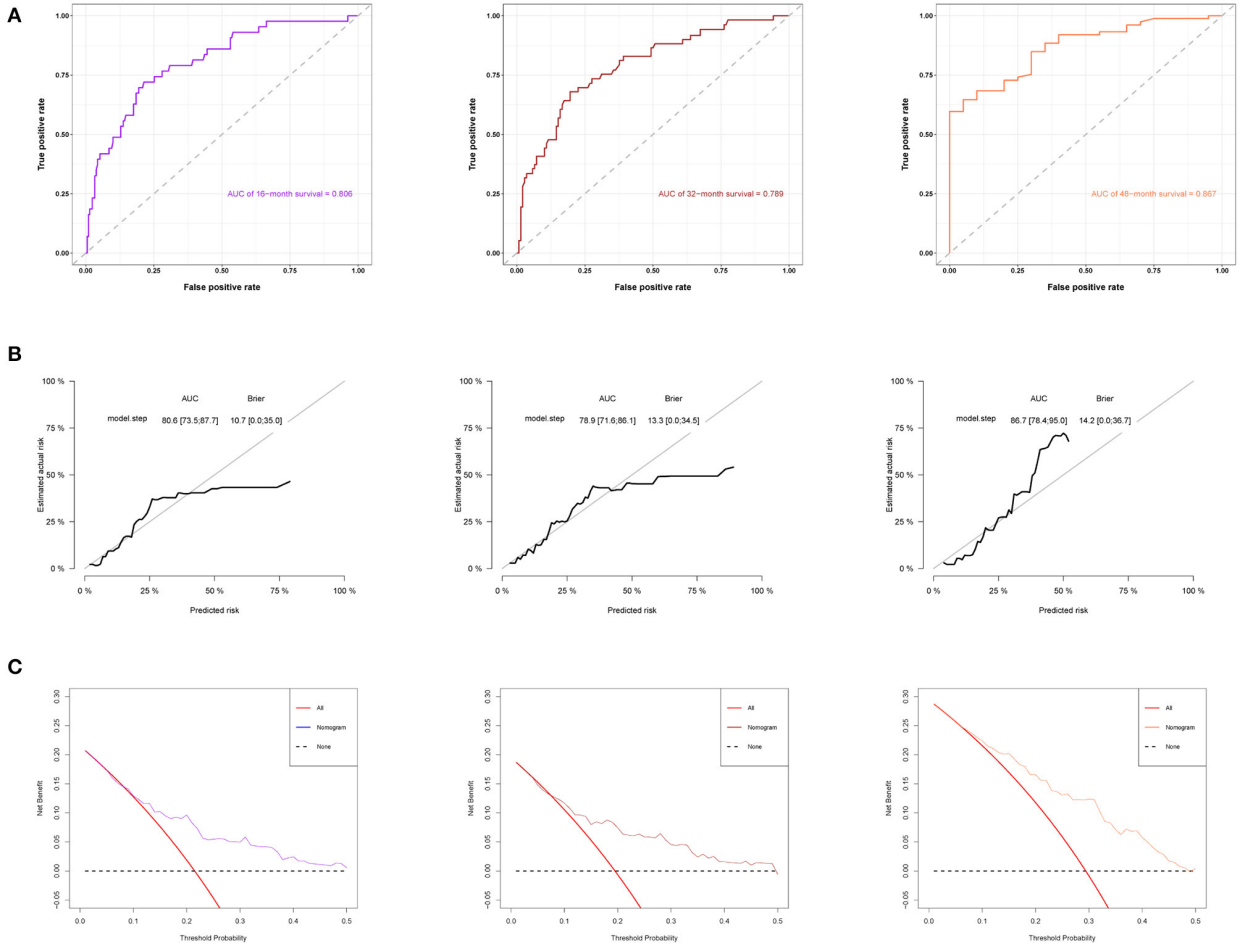
Model discrimination and performance in the validation set. **(A)** ROC curves for nomogram-based prognostic prediction. **(B)** Calibration curve of 16-, 32-, and 48-month specific survival. **(C)** DCA of this nomogram in the validation set.

We used the validation cohort to evaluate the predictive models. The discrimination, calibration, and clinical effectiveness of nomograms were evaluated using the ROC curves, calibration curves, and DCA, respectively. Model 2 showed good discrimination for prognostic prediction, with AUCs of 0.806, 0.789, and 0.867 for the 16-, 32-, and 48-month survival rates, respectively (Figure 5A). In addition, we performed a calibration curve analysis, whose results showed that the model had good probabilistic agreement between the predictions and observations on the validation set (Figure 5B). The model was better at predicting DOC survival (Figure 5C).

### Risk Stratification by Nomogram

**Figure 6:**
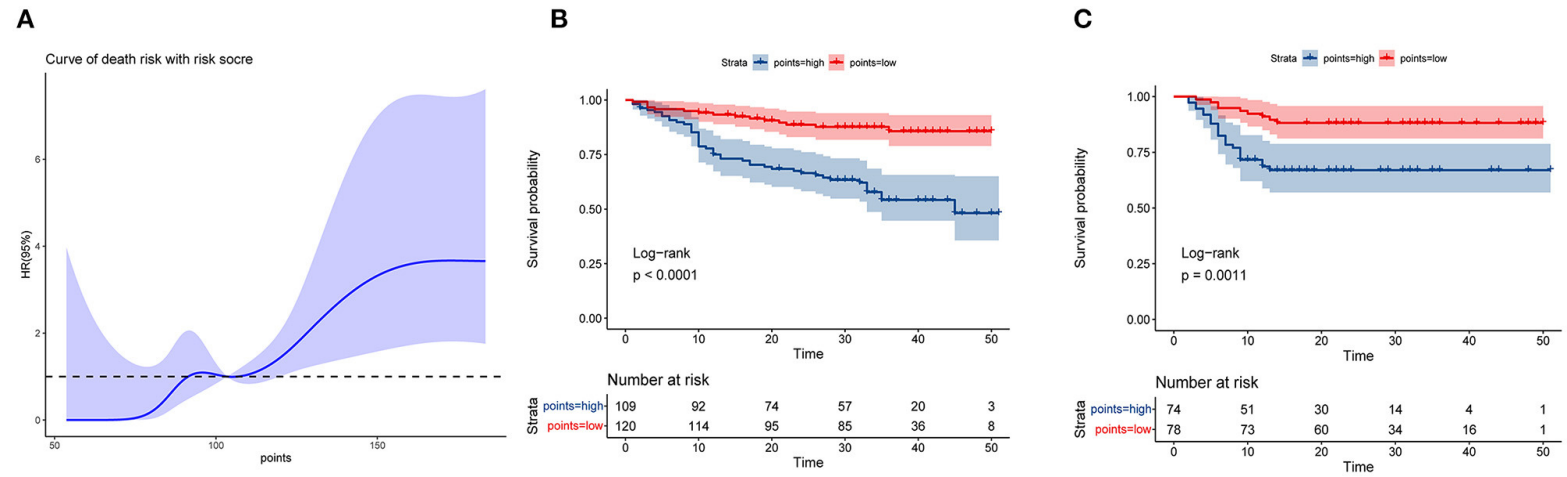
Performance of the nomogram in stratifying the risk of patients. **(A)** Restricted cubic spline analysis of the total risk score in the training set. **(B)** Kaplan–Meier survival analysis between the low- and high-risk groups in the training set. **(C)** Kaplan–Meier survival analysis between the low- and high-risk groups in the validation set.

For the training set, the nomogram was used to calculate the total prognosis score. Classification was performed using a restricted cubic spline assessment (Figure 6A), and the total prognostic scores were divided into two risk groups to predict mortality (group A: 0–100 points, group B: >100 points). For both the training and validation sets, the survival analysis of patients with low-risk disease showed significantly better outcomes than those with high-risk disease (*p* < 0.0001 and *p* = 0.0011; Figures 6B,C).

## Discussion

With the continuous improvement of current diagnosis and treatment systems, the mortality rate of high-risk diseases, such as traumatic brain injury and cerebral apoplexy, has been declining, but the number of patients with DOC tends to increase. Predicting the survival time of patients with DOCs will aid in the development of appropriate healthcare and DOC management guidelines and will be highly beneficial for clinicians in personalizing DOC management and optimizing limited health resources. Through a retrospective study of DOC patients in two centers, we comprehensively evaluated the relationships between routine clinical scores, laboratory tests, and DOC mortality rates. An optimal predictive model for predicting DOC-related mortality with satisfactory consistency and accuracy was successfully developed and carefully evaluated.

Available evidence suggests that the prognosis of patients can be judged simply and intuitively from a nomogram, which makes it convenient for clinical personnel to use and interpret its results (Lei et al., 2016; Chen et al., 2019). To the best of our knowledge, our current model is the first to be used to develop a nomogram to predict DOC survival probability using simple and readily available metrics. We developed a multivariate Cox proportional hazards regression model (model 2) based on our observation that age, GCS score, midline shift, and low albumin concentration were independently associated with mortality related to DOC. In addition, there was no clear association between consciousness status and death outcome in our multivariate Cox proportional hazards regression model. However, we included them in model 1 based on clinical evidence. By comparing the areas under the ROC curves of models 1 and 2, we finally settled with model 2 as our final model. Model 2 was based on four features (age, GCS, midline shift, and albumin concentration), and it showed good discriminative ability for both the training and validation sets. For the training set, the AUCs for the 16-, 32-, and 48-month survival rates were 0.791, 0.760, and 0.886, respectively. For the validation set, the AUCs for the 16-, 32-, and 48-month survival rates were 0.806, 0.789, and 0.867, respectively. Model performance was also evaluated using calibration curves and DCA for both the datasets. Our results suggest that the model can serve as a cost-effective tool to predict the prognosis of DOC and assist in clinical decision-making.

So far, few studies have been done on the markers of survival outcomes in patients with DOC. Therefore, the variables selected are the most readily available and easy to ascertain for widespread clinical application. For our model, we identified four variables for the survival prediction of DOC. First, age had the largest strongest association. Previous studies have shown that prognosis deteriorates with age in patients with DOC. The possible reason is that meningeal fibrosis is more severe in older patients than in young patients due to the decline in circulatory and metabolic capacity with age. This affects the circulation and absorption of cerebrospinal fluid, in addition to the significantly weaker resistance to disease progression than in younger patients (Hao et al., 2016). Therefore, age is considered the greatest risk factor for DOC mortality.

The second variable is the GCS score, which is one of the most widely used tools for examining a patient's level of consciousness (Perel et al., 2008; Dijkland et al., 2019). Our study showed a significant correlation between the GCS score and patient survival outcomes, which is consistent with previous reports. Our third variable, brain midline shift, refers to changes in the position of the midline structures of the brain, which are closely related to the hypothalamic-pituitary axis, the efferent and afferent pathways, and the centers that regulate important life activities such as breathing and heart rate. Brain midline shift can lead to abnormalities in the frequency, rhythm, and amplitude of the heartbeat and respiration of patients. In addition, the patient may also experience endocrine dysfunction, which is a serious threat to life and causes a poor prognosis. Therefore, in our study, we found that the survival outcomes of patients with a midline shift were significantly different from those without it. The fourth variable is hypoproteinemia. This is not an independent disease, but a negative nitrogen balance due to various reasons. Albumin accounts for two-thirds of the total plasma protein and plays an important role in the transport and binding of many molecules. Patients with pDOC may have low serum albumin concentrations because of the following causes (Corrigan et al., 2016; Sorby-Adams et al., 2017). First, bleeding: trauma leads to vascular damage, which may involve the blood-brain barrier, which causes loss of albumin and hemoglobin. Second, the strong stress after traumatic brain injury may also lead to the release of inflammatory mediators, thereby enhancing vascular permeability. This may promote the transfer of albumin from the blood vessels to the interstitial space. Hypoalbuminemia can seriously impair the defense and immune function of patients with DOC and cause various complications such as pulmonary infection (Sung et al., 2004), which seriously affects the prognosis of DOC.

Predicting the outcome of patients with DOC is challenging. Joint prediction of multiple variables can reduce errors and improve overall accuracy. At present, in the neuroscience community, the most explored brain monitoring technologies are fMRI, positron emission tomography, and EEG (Stender et al., 2014; Wang et al., 2015; Song et al., 2020). However, these technologies are not only difficult to operate but also expensive, which makes them prohibitive for medical institutions of different levels. Furthermore, there are currently no multivariate models to predict mortality outcomes.

The prediction model established in this study is based on four clinical parameters. Previous studies have shown that the level of consciousness and CRS-R score are correlated with the prognosis of DOC (Giacino et al., 2018). However, in this study, the CRS-R score showed no significant difference in either univariate or multivariate Cox regression, while the consciousness status was only statistically different for univariate Cox analysis, which may be related to its heterogeneity. Our study has several important characteristics. First, the nomogram is a model with the ability to predict prognosis that integrates meaningful variables, provides graphs and visualizations of data, and predicts individual outcomes in a very detailed and intuitive way. We distributed our nomogram via a website, which is convenient for clinical use. Second, previous studies have focused on predicting the probability of improvement in DOC, and this study is the first to develop a model to predict DOC survival outcomes. Our study provides foundational insights for clinical decision-making of patients with DOC. In addition, our model used clinical variables that are easy to obtain. Through verification, we found that this model had good clinical application value.

This study had limitations. First, it was a retrospective cohort study involving a small sample. Therefore, it was subject to information bias and selection bias, which is also the inherent limitation of retrospective research. To validate our model, it is necessary to carry out a prospective study with a larger sample size in multiple centers. Second, GCS and albumin concentration in our model may not have been stable throughout the follow-up period, which may have affected the accuracy of prediction to an extent. Third, the characteristics of patients with impaired consciousness may differ with medical settings and regions. Our study participants were exclusively Chinese and limited to Jiangxi Province, which may limit the generalizability of our findings to a wider population. Therefore, it is still necessary to select multicenter experiments in different regions to verify the accuracy and effectiveness of the model.

In conclusion, our study identified age, GCS score, CT midline shift, and albumin concentrations as significant predictors of the survival outcomes of DOC. A website based on a nomogram was developed to use this model easily in clinical settings. In addition, as the model can distinguish the patients with DOC who have a high risk of mortality, it is useful for use during the follow-up periods. This model may facilitate decision-making, preventive strategies, and individualized treatment for DOC.

## Data Availability Statement

The datasets generated or analyzed during this study are available from the corresponding author on reasonable request.

## Ethics Statement

The studies involving human participants were reviewed and approved by Ethics Committee of the First Affiliated Hospital of Nanchang University. The patients/participants provided their written informed consent to participate in this study.

## Author Contributions

JK conducted experiments and wrote manuscripts. YZ and GC designed the study and collected the data. LH performed the statistical analysis of the data. YT and WY searched the relevant literature. ZF secured funding for the project. All authors have read and approved the final manuscript.

## Funding

The study was funded by the Major Research Development Program of Jiangxi Province (Grant No. 20202BBG72002), National Natural Science Foundation of China (Grant No. 82160437), Youth Talent Cultivation Project of First Affiliated Hospital of Nanchang University (Grant No. PRJ-20211017170929794), and Science and Technology Project of Jiangxi Provincial Health Commission (Grant No. 202210362).

## Conflict of Interest

The authors declare that the research was conducted in the absence of any commercial or financial relationships that could be construed as a potential conflict of interest.

## Publisher's Note

All claims expressed in this article are solely those of the authors and do not necessarily represent those of their affiliated organizations, or those of the publisher, the editors and the reviewers. Any product that may be evaluated in this article, or claim that may be made by its manufacturer, is not guaranteed or endorsed by the publisher.
